# Plant-Based Strategies for Vaccine Development: A Narrative Review of Recombinant Biofactories, Phytochemical Adjuvants, Innovative Delivery Systems, and Insights on Oral and Edible Vaccines

**DOI:** 10.3390/vaccines14050391

**Published:** 2026-04-27

**Authors:** Kianoosh Najafi, Maryam Jojani, Soroosh Najafi, Giovanni N. Roviello

**Affiliations:** 1 School of Medicine and Surgery, University of Naples ‘Federico II’, Via S. Pansini 5, I-80131 Naples, Italy; 2 Scientific and Production Center “Armbiotechnology” NAS RA, 14 Gyurjyan Str., Yerevan 0056, Armenia; 3 Institute of Biostructures and Bioimaging (IBB), CNR National Research Council of Italy, I-80145 Naples, Italy

**Keywords:** plant-based vaccines, recombinant antigens, microalgae, phytochemical adjuvants, oral vaccine delivery, immunostimulants, phytocompounds

## Abstract

**Background/Objectives:** Vaccination is a critical public health intervention, yet its global implementation is hindered by high production costs and cold-chain requirements. This review aims to evaluate plant-based systems as sustainable, cost-efficient alternatives for vaccine production. **Methods**: A comprehensive literature search was conducted across major databases (PubMed, Scopus, Web of Science). The peer-reviewed references were critically assessed, focusing on molecular expression strategies, phytochemical immunomodulators, and plant-mediated oral delivery. **Results**: Plant and microalgae systems effectively support nuclear, chloroplast, and transient expression of diverse antigens. Furthermore, specific plant-derived compounds were found to act as potent adjuvants and immunostimulants, enhancing the immunogenicity of vaccine formulations. Edible plant tissues also provide a viable platform for oral delivery, reducing the need for extensive purification and refrigerated logistics. **Conclusions**: Integrating recombinant expression technologies with bioactive plant metabolites offers a flexible and scalable foundation for next-generation vaccines. These biological platforms show promise for addressing some immunization challenges, particularly in low-resource settings.

## 1. Introduction

Vaccination has long served as one of the most effective public health interventions, limiting the spread of infectious diseases and, in some regions, contributing to their eradication [1]. Despite their proven value, several practical constraints continue to restrict broader implementation. Key challenges include the identification of appropriate antigenic targets, the development of cost-efficient production systems, and the establishment of accessible delivery formats [2,3]. Oral immunization offers potential solutions to several of these barriers. By eliminating the need for injections, oral formulations reduce dependence on trained medical personnel and diminish the requirement for highly purified material [4,5]. Needle-free administration [4] also improves acceptability, particularly among children, and can enhance vaccine accessibility in low-resource settings by reducing reliance on cold-chain infrastructure. These factors collectively support improved compliance across diverse populations. The success of the oral polio vaccine illustrates the feasibility of large-scale oral immunization [6]. Nevertheless, most contemporary vaccine candidates remain injectable [7], largely due to unresolved technical obstacles associated with oral delivery. Effective oral vaccines must withstand degradation in the gastrointestinal tract, necessitating substantially higher antigen quantities than parenteral formulations. As a result, production demands and raw material costs increase dramatically, highlighting the difficulty of achieving both low cost and high volume. These constraints become even more pronounced when recombinant DNA technologies are used to generate subunit vaccines [8]. Although subunit antigens offer strong safety profiles, their expression outside the native pathogen can be inefficient. Mammalian cell cultures provide appropriate post-translational modifications but are prohibitively expensive for the large-scale output required for oral delivery [9]. Transgenic animals have been explored as an alternative, particularly through expression in milk, but significant practical and regulatory challenges remain unresolved [10].

Plant-based expression systems have emerged as promising recombinant biofactories for producing therapeutic proteins and vaccine antigens [11]. Plants provide several advantages: the capacity for eukaryotic post-translational modifications, rapid scalability, low production costs, and minimal risk of contamination with human pathogens. When edible plant tissues are used as delivery vehicles, purification steps may be reduced or eliminated, enabling oral administration in a safe and palatable form [12]. Grain crops offer additional benefits through ambient-temperature stability, potentially removing cold-chain requirements. Moreover, plants can accommodate the simultaneous expression of multiple transgenes, enabling multivalent vaccine formulations within a single product [12]. These features suggest that plant-based systems could become attractive platforms for developing oral vaccines with improved accessibility and reduced manufacturing constraints. Plant-based expression platforms have demonstrated the capacity to generate a wide range of subunit vaccine antigens. Multiple expression strategies, spanning nuclear, chloroplast, and transient systems, have been employed to produce diverse vaccine candidates. Numerous plant-derived subunit antigens have been purified and shown to induce measurable immune responses following parenteral administration in animal models. In addition to purified formulations, several vaccine candidates have been delivered orally in unprocessed plant tissues used as food or feed, where orally ingested plant material has been shown to stimulate immune responses and, in some instances, provide protective effects [12].

Beyond plant-derived vaccine platforms, progress across the wider biomedical landscape further contextualizes the technological and conceptual advances driving current therapeutic innovation. In fact, biomedicine continues to progress on multiple fronts, among them the creation of supramolecular systems like metallogels designed for therapeutic use [13], as well as ongoing investigations of natural compounds in the context of neurodegenerative disorders [14]. Further advances have been achieved in clarifying the mechanisms of intracellular parasitic and viral diseases and assessing their impact [15,16], along with advances in precision therapies driven by bioactive molecules, multi-omics approaches, and drug-repurposing frameworks [17]. Moreover, recent research has advanced our understanding of protein folding in disease [18], and enabled the design of new anti-infective scaffolds [19,20,21]. These developments are supported by progress in biomacromolecular dynamics [22,23,24,25,26,27,28,29,30,31], computer-assisted biological analysis [32,33], and nature-inspired agents targeting disease-related proteins [34,35], highlighting the broad innovation shaping modern biomedical science. These advances further involve the valorization of agri-food residues for the creation of functional products [36,37,38,39,40,41,42,43,44,45,46], as well as advances in analytical-chemistry techniques [47,48]. Together, these areas delineate the scientific and technological foundations examined in this comprehensive review, which surveys plant-based vaccine production alongside emerging biomedical strategies that could contribute to reshaping modern immunoprophylaxis, including the use of plant-derived compounds with intrinsic immunostimulatory or adjuvant properties.

## 2. Plant-Based Expression Platforms for Recombinant Vaccine Antigen Production

This section highlights the expanding role of plant-derived platforms in next-generation vaccine development, encompassing antigen production, immune-enhancing molecules, and innovative delivery strategies, as detailed in the following subsections.

### 2.1. Plant-Based Vaccines: Plants as Biofactories and Cellular Platforms for Antigen Production

Plant expression systems are being explored as potentially scalable and cost-efficient biofactories, although industrial-scale validation remains limited.

#### 2.1.1. Genetic Transformation and Vector Systems

Antigen production begins with the introduction of a gene of interest into the plant cell, typically with two main mechanisms. In stable (transgenic) systems, the antigen gene is permanently integrated into the plant’s nuclear or plastid genome. In this system, it takes months to years to select viable transgenic lines and grow complete plants from tissue culture. However, once established, it produces a consistent expression of the antigen throughout all the plant tissues. It can be propagated by seeds, and successive generations maintain antigen production [49,50]. Chloroplast transformation offers a high-yield alternative to nuclear integration. Because each plant cell contains hundreds to thousands of chloroplasts, and each chloroplast contains multiple copies of its genome, the gene copy number can reach up to 10,000 per cell [51]. This over-expression can lead to antigen accumulation levels exceeding 70% of the leaf’s total soluble protein [52]. Furthermore, plastid transformation is associated with increased biosafety because chloroplasts are mostly maternally inherited, and the transgene is not present in pollen, thereby decreasing the risk of gene flow for non-target crops [53]. However, unlike nuclear transformation, plastids do not support mammalian-like *N*-glycosylation and are therefore best suited to non-glycosylated antigens or oral bioencapsulated formats [54]. The second mechanism, transient expression, delivers the antigen gene into plant cells without chromosomal integration and permanent changes in the host genome [55]. In transient expression, plant cells can be programmed to produce the target protein within a matter of days, making it excellent for responding to emerging health threats [56]. *Nicotiana benthamiana* is widely used for transient expression because it is highly susceptible to *Agrobacterium* and viral replicons. The agroinfiltration method uses *Agrobacterium tumefaciens* bacteria as a delivery vehicle to introduce genes into plant cells [57]. The gene of interest is cloned into a binary vector within the bacterium’s Ti plasmid [58]. Syringe infiltration is simple and flexible, and allows for the infiltration of one or multiple constructs on the same leaf. Vacuum infiltration involves submerging whole plants in *Agrobacterium* solution under vacuum so the intercellular air is replaced by the suspension. However, this method is more technically complex but enables very large-scale batches [59]. Once the infiltration is complete, the bacteria transfer their T-DNA into the plant cell nuclei. The transferred DNA remains episomal rather than integrating into the host chromosome, and transiently expresses over several days before it is gradually diluted as cells divide [60]. In another method using viral vectors, genes encoding viral movement, coat protein synthesis, and other potentially harmful structures are removed, and the transgene encoding the vaccine antigen is inserted. Commonly used plant virus vectors include Tobacco mosaic virus (TMV), Cowpea mosaic virus (CPMV), and Potato virus X (PVX) [61]. This method results in high-level protein accumulation and minimizes biosafety risks associated with infectious agents [62].

#### 2.1.2. Transcriptional and Translational Control

At the transcriptional level, strong plant promoters lead to high mRNA production. Constitutive promoters such as the Cauliflower mosaic virus (CaMV) 35S promoter, a widely used viral promoter, or the ubiquitin promoter are commonly used to achieve this goal [63]. However, these cause mRNA production in all tissues, whereas tissue-specific or inducible promoters are valuable when spatial and temporal control is needed. For example, a fruit-specific promoter was used to restrict expression of an oral vaccine antigen against enterotoxigenic *Escherichia coli* (ETEC) to tomato fruits, while seed-specific promoters enabled the accumulation of antigens targeting pathogens such as hepatitis B virus (HBV) and cholera toxin in rice grains for oral vaccine prototypes [64,65]. Inducible promoter systems triggered by chemicals, stress, or light enable antigen expression at a desired time [66]. In addition, regulatory genetic elements from plant viruses can increase transcription and mRNA levels [67]. At the translational stage, optimizing codon usage and mRNA stability are important for maximizing antigen yield. Genes derived from humans or pathogens often contain codons that are less preferred by plants, which can lead to ribosomal stalling and translational pauses [68]. To prevent this, the coding sequences of vaccine antigens are typically codon-optimized, and rare codons are replaced with synonymous “preferred” codons that match the plant’s most abundant tRNAs [69]. Furthermore, engineered non-coding regions can improve translation efficiency; for example, the inclusion of potent 5′ UTR leaders from highly expressed genes, such as the Tobacco mosaic virus Ω sequence, and appropriate 3′ UTRs can improve ribosome recruitment and mRNA stability. Additionally, plants can recognize introduced transgenes and produce small interfering RNAs that suppress mRNA accumulation. This effect can be minimized by co-expressing viral silencing suppressor proteins, such as tombusvirus P19 or potyvirus HC-Pro, alongside the antigen gene [70]. For example, influenza vaccine antigens have been produced in *Nicotiana benthamiana* using transient expression systems that contain silencing suppressors. In one study, co-expression with P19 supported high-level accumulation of H7 hemagglutinin, with peak expression detected at 6 days post-infiltration, and the purified protein maintained hemagglutination and hemagglutination-inhibition activity [71]. More generally, launch-vector systems that include silencing-suppression strategies have been used to rapidly produce high levels of influenza antigens in plants [72].

#### 2.1.3. Glycoengineering and Humanized Glycosylation in Plant Systems

The conserved *N*-glycan core (Man_3_GlcNAc_2_) is shared across eukaryotes. However, plant-specific modifications include the addition of β-1,2-linked xylose and core α-1,3-linked fucose, which are absent in humans, are potentially immunogenic, and may lead to the formation of anti-plant glycan antibodies [73]. Conversely, human glycans are characterized by the addition of α-1,6-linked fucose and terminal extensions such as β-1,4-linked galactose and *N*-acetylneuraminic acid (sialic acid), which are not naturally synthesized by plants. The primary step in humanizing plant glycosylation is the knockout or downregulation of the endogenous β-1,2-xylosyltransferase and α-1,3-fucosyltransferase genes [74]. Using methods such as CRISPR/Cas9, RNAi, or gene targeting via homologous recombination, this has been successfully done in a number of plant systems, including *Nicotiana benthamiana* and the moss *Physcomitrella patens* [75,76]. Furthermore, achieving full humanization requires the terminal addition of sialic acid, which is achieved by expressing mammalian glycosyltransferases (e.g., β-1,4-galactosyltransferase) and installing a multi-gene pathway that enables Neu5Ac biosynthesis, activation to CMP-Neu5Ac, Golgi transport, and transfer to terminal galactose [77,78].

Practical examples of glycoengineered plant systems include HIV-1 gp120/gp140 produced in *Nicotiana benthamiana,* which carry human-like complex *N*-glycans with terminal sialylation and keep binding to CD4 and neutralizing antibodies [79]. Furthermore, monoclonal antibodies produced in plants with engineered glycosylation pathways display terminal galactosylation and sialylation while retaining antigen binding and Fc-mediated activity [78]. Likewise, recombinant human erythropoietin produced in glycoengineered plants shows sialylated glycoforms with improved circulatory stability [80].

#### 2.1.4. Subcellular Targeting and Protein Folding

The intracellular destination of a recombinant antigen in plant cells dramatically improves its stability and assembly. Adding an *N*-terminal signal peptide to the gene directs the antigen to the secretory pathway and guides the nascent protein into the endoplasmic reticulum (ER), where it is cleaved off, and the protein can fold with the assistance of ER chaperones and oxidizing conditions, such as disulfide bond formation [81]. The protein then enters the Golgi apparatus and can be secreted to the apoplast unless an ER-retention signal is used. Targeting recombinant proteins to the ER/Golgi has promoted correct tertiary/quaternary folding and enables post-translational modifications such as *N*-glycosylation that are often essential for antigen function or immunogenicity [82]. However, if the proteins are secreted to the apoplast, the abundance of endogenous proteases can degrade them. To prevent this, a common tactic is to include a *C*-terminal ER-retention motif (such as KDEL) on the antigen, which causes it to be retained in the ER [83]. Another option is targeting the vacuole or other storage organelles. There are different kinds of plant vacuoles. In leaves, they are usually lytic and have active degradative enzymes, which are less ideal for sensitive proteins, whereas in seeds, they commonly function as protein storage vacuoles and can keep proteins in a stable, inert environment [84]. Notably, the first Food and Drug Administration (FDA)-approved plant-derived pharmaceutical, taliglucerase alfa, used for Gaucher disease, was produced in carrot cells by targeting the enzyme to vacuoles, where plant-specific mannosidase activity generated the desired terminal mannose glycan on the enzyme [85]. For vaccine antigens that do not require glycosylation, chloroplasts, thanks to the organelle’s high genome copy number and protein-synthesis capacity, can yield greater amounts and retain immunogenicity [86].

#### 2.1.5. Virus-like Particles (VLPs)

These particles are composed of viral structural proteins that mimic the size and shape of a real virus but lack the genetic material required for replication. Their production approaches can be divided into non-enveloped capsid VLPs (e.g., HPV L1, norovirus VP1) and enveloped VLPs that have a lipid bilayer derived from the host cell membrane (e.g., influenza HA VLPs; SARS-CoV-2 spike-displaying CoVLP) [87]. Interestingly, plants can produce chimeric particles where a foreign antigen is displayed on the surface of a carrier VLP, such as the hepatitis B core antigen (HBcAg) on the Cowpea mosaic virus (CPMV) [88]. The assembly process is directed by the spontaneous interactions along with the polymerization of expressed proteins within the ER or cytoplasm, depending on the targeting signals used [87]. The structure of VLPs (typically 20–300 nm in diameter) is ideal for recognition by antigen-presenting cells (APCs), particularly dendritic cells [89]. The repetitive display of epitopes on the VLP surface can also directly cross-link B-cell receptors and result in high antibody titers and long-lasting B-cell memory [90]. Furthermore, VLPs are efficiently processed for presentation to both MHC class I and class II molecules and elicit robust cytotoxic T-lymphocyte responses [91]. In addition to structural benefits, plant-derived VLPs have shown immunogenicity and protective efficacy. For example, SARS-CoV-2 VLP vaccines developed in *Nicotiana benthamiana* (e.g., Medicago CoVLP) have reached clinical trials and induced neutralizing antibody titers and antigen-specific IgG responses, along with balanced cellular immunity represented by both Th1 (IFN-γ) and Th2 (IL-4) responses. Also, clinical studies have indicated protection against symptomatic infection and severe disease [92,93].

Similarly, plant-derived influenza VLPs expressing hemagglutinin (HA) have been shown to trigger high levels of neutralizing antibodies, as measured by hemagglutination inhibition assays, alongside the activation of both CD4+ and CD8+ T-cell responses. In animal models, these vaccines reduced viral loads, lightened weight loss, and improved survival rates, confirming their protective efficacy. Chimeric VLP platforms, particularly those based on hepatitis B core antigen (HBcAg), can also present heterologous epitopes and stimulate humoral immune responses [94].

#### 2.1.6. Downstream Processing and Purification Strategies

Downstream processing (DSP) is the major economic bottleneck in the plant-made vaccines production and can account for 80% or more of total manufacturing costs [95]. Leaf tissues contain impurities, including phenolics, pigments, proteases, lipids, polysaccharides, and nucleic acids, which make purification difficult and result in reduced yields and increased costs [70]. As a result, upstream pathways must be designed in a DSP-compatible antigen format, including subcellular targeting, secretion, and glycoengineering [96]. In whole-plant extraction, leaves are harvested and subsequently disrupted, either mechanically or enzymatically, before proceeding through the downstream purification steps to clarify the extract. For example, Covifenz production includes enzyme-assisted extraction, low pH treatment, coarse filtration, centrifugation, depth filtration, and microfiltration prior to chromatography [97]. In contrast, plant cell suspension cultures are more consistent at the start of the process [98]. When recombinant proteins are released into the culture medium, they are easier to collect, and downstream processing costs are lower [99]. Due to high solids and impurities that clog filters and resins, clarification in plant systems is challenging [100]. Early steps must minimize oxidative browning from phenolics and prevent protein breakdown [97]. After clarification, purification proceeds through capture (usually ion exchange), polishing (removal of aggregates, host cell proteins, and DNA), and buffer exchange (ultrafiltration/diafiltration) [101]. Affinity tags are helpful during development, but if they need to be removed, they can complicate large-scale production [96]. Additionally, some plants require customized DSP due to plant-specific contaminants, such as nicotine in *Nicotiana* species [97]. Safety strategies against viruses differ from those against mammalian-derived materials. Some plant-based VLPs do not meet the usual standards for virus clearance. Instead, controlling raw materials, monitoring the environment, and conducting extensive testing ensure safety [89]. Overall, plant DSP needs process engineering that accounts for impurities and an integrated upstream-downstream design to achieve maximum safety [102]. An overview of representative plant-based vaccine platforms and their development status is presented in Table 1.

Among the various plant-derived antigens evaluated to date, the cholera toxin B subunit (CTB, Table 1) represents a leading example, with the MucoRice-CTB platform demonstrating clinically validated oral delivery and highlighting the unique advantages of seed-based expression systems.

### 2.2. Microalgae-Derived Vaccines

Although microalgae share fundamental biological characteristics with plant-based vaccine platforms, they have significant differences. First, cultivation is inherently bioreactor or pond-based, which allows for physical containment, batch-to-batch control, and quick scale modulation without the planting and harvesting logistics that come with whole plants. The study on the *Chlamydomonas* CTB-D2 vaccine specifically used confined Wave Bioreactor cultivation to grow biomass for oral administration [120]. Second, microalgae support a spectrum of production modes, including photoautotrophic growth (light-driven CO_2_ fixation with minimal external carbon inputs), mixotrophy (simultaneous use of light and organic carbon sources), and, in some cases, heterotrophic/fermentation-style growth (light-independent cultivation using organic substrates in closed bioreactors). For *Chlamydomonas reinhardtii*, fermentation-compatible biomass productivities were explored in the context of commercial feasibility for recombinant antigens [121]. Third, microalgal cells can function simultaneously as a manufacturing chassis and an oral delivery matrix. Freeze-dried biomass can protect antigens during storage and partially through gastric transit (bioencapsulation) [120,122]. Fourth, post-translational modifications (PTMs) diverge from both higher plants and mammalian cells. Eukaryotic microalgae can install *N*-glycans, but the structures are often non-canonical (e.g., methylation and plant-like core xylose/fucose features in *Chlamydomonas*), and chloroplast-expressed proteins (such as in many proof-of-concept oral vaccines) are typically non-glycosylated [123]. This makes antigen selection and compartment targeting unusually consequential for clinical translation.

#### 2.2.1. Nuclear vs. Chloroplast Expression in Microalgae

In *Chlamydomonas*, chloroplast expression has been repeatedly used for oral vaccine prototypes because it offers stable integration and high accumulation of properly folded, disulfide-rich proteins for antigens that do not require *N*-glycosylation. For example, fusion antigens such as CTB-VP1, consisting of the cholera toxin B subunit fused to the VP1 capsid protein of foot-and-mouth disease virus [124], and CTB-D2, in which CTB is fused to the D2 domain of the *E. coli* heat-labile enterotoxin [120], are two well-known examples of chloroplast engineering that enabled stable expression suitable for whole-cell oral delivery. However, chloroplast expression can be incompatible with glycosylated antigens and can introduce antigen processing. In Berndt et al. (2021), a chloroplast-localized RBD fusion was truncated and not recognized as intended, whereas ER-retained or secreted versions accumulated intact and were functional in binding assays with the SARS-CoV-2 cell receptor, angiotensin-converting enzyme 2 (ACE2) [121]. This illustrates compartment choice, chloroplasts for the yield/stability of aglycosylated targets, and secretory routing for glycoproteins and receptor-binding authenticity. In *Nannochloropsis*, the principal high-yield demonstration for a vaccine antigen, VP2, the major capsid protein of Infectious Bursal Disease Virus (IBDV), was achieved through nuclear expression [125], and efficient nuclear promoter architectures have been shown to bridge the gap between chloroplast and nuclear expression yields in some algae.

#### 2.2.2. Transient/Viral-Vector and Episomal Systems

Microalgal transient expression has advanced via plant-virus-derived replicons adapted to microalgae. The “Algevir” geminiviral vector concept was introduced as an inducible Rep-mediated replication system enabling high-level expression of antigenic proteins in microalgae, including Zaire ebolavirus GP1 and the B subunit of heat-labile *E. coli* enterotoxin (LT-B), both retaining antigenic activity [126]. Furthermore, limitations, especially associated with *Agrobacterium tumefaciens*-mediated transformation, particularly the risk of residual bacterial contamination that may compromise oral formulations, have motivated the development of viral-vector approaches to avoid bacterial carryover. Episomal tools are also increasingly relevant, especially for enabling editing without leaving selectable markers or other foreign DNA permanently integrated in the host genome, or for reversible expression. For instance, episomal CRISPR systems have been reported in *Nannochloropsis* [127], and a more recent base-editing approach uses an episomal vector backbone (centromere + autonomous replication sequence) in *N. oceanica* to enable subsequent plasmid loss after editing [128].

#### 2.2.3. Promoters, UTRs, and Selectable Markers

Microalgae vaccines often encode the regulatory architecture of transgene expression in UTRs and endogenous regulatory elements, rather than relying on the classic plant promoter framework [129]. Examples include chloroplast expression using photosynthesis-gene UTRs (e.g., rbcL UTR control of CTB-D2 in *Chlamydomonas chloroplasts* [120]; and chloroplast integration of CTB-VP1 by biolistics [124]). Selectable markers in chloroplast systems frequently rely on antibiotic resistance cassettes such as aadA (spectinomycin/streptomycin) to enable selection of successfully transformed lines under antibiotic pressure [130]. At the nuclear level, promoter and leader selection can dominate yield by orders of magnitude across transformants. In *N. oceanica*, Rout et al. (2022) explicitly compared endogenous promoters using qPCR/flow cytometry and achieved the highest VP2 yields with the elongation factor promoter with enhancer effects from its *N*-terminal leader sequence [125]. They also observed transformant-to-transformant production differences spanning orders of magnitude, with multiple vector integrations correlating with peak yields [125]. In *Chlorella*, an oral vaccine study targeting VP28, an envelope protein of White Spot Syndrome Virus (WSSV), used a codon-optimized cassette integrated via homologous recombination at the nitrate reductase locus and driven by a CaMV 35S promoter with a *Chlamydomonas* RbcS2 terminator, an example of cross-kingdom regulatory borrowing for practical expression [89].

#### 2.2.4. Glycosylation and PTMs in Microalgae

For secreted/ER-routed antigens, microalgal *N*-glycosylation can determine antigen folding, receptor binding, and immunogenicity. In *C. reinhardtii*, studies show endogenous proteins carry predominantly oligomannosides (Man-2 to Man-5), with minor complex *N*-glycans that can be partially *O*-methylated and include xylose residues [131]. Subsequent work identifies multiple xylosyltransferases contributing to heterogeneous *N*-glycan xylosylation, including core β-(1,2)-xylose features and methylation, and genetic studies continue to map enzymes affecting core fucosylation and xylosylation (e.g., identification of candidate core xylosyltransferase XylT1B) [132]. Heterologous expression of GnTI has been used to probe and further explore microalgal *N*-glycosylation capacity [133]. From a vaccine development point of view, this means that antigens that are meant to mimic mammalian glycoepitopes may need to be targeted to the secretion pathway and have glycoengineering and analytics plans [129]. On the other hand, antigens whose protective epitopes are glycan-independent may be more effectively produced through chloroplast/cytosolic expression and oral bioencapsulation to make them stronger and less complicated [134].

#### 2.2.5. Downstream Processing

As with plant-based vaccines, downstream processing also constitutes a significant economic bottleneck in microalgal vaccine production [135]. A key challenge specific to microalgae is the presence of a rigid, cellulose-rich cell wall, which necessitates efficient disruption methods to release intracellularly produced vaccine antigens [136,137].

The first step in DSP is harvesting microalgal biomass from dilute cultures. Once harvested, the rigid cell wall of microalgae must be disrupted to release intracellularly produced vaccine antigens. High-pressure homogenization (HPH) is particularly suitable for large-scale operations, forcing cell suspensions through a narrow orifice at high pressure to generate shear forces that rupture the cell wall. Ultrasonication, on the other hand, utilizes acoustic cavitation to fragment the cell membrane [138]. Studies have shown that HPH can have a disruption rate constant approximately seven times higher than that of ultrasonication, making it the preferred choice for high-volume commercial production [139]. In addition to common impurities such as host cell proteins (HCPs) and DNA, microalgal extracts contain high levels of pigments (e.g., chlorophyll and carotenoids) and polysaccharides, which can complicate downstream purification. Therefore, additional clarification and purification steps are often required [140].

The actual purification of the recombinant antigen typically involves a combination of filtration and chromatography. Size-exclusion chromatography (SEC) and anion-exchange chromatography (AEC) are frequently used to remove process-related impurities, such as host cell proteins (HCPs) and DNA [141]. Emerging technologies, such as steric exclusion chromatography (SXC) using low-cost stationary phases, such as cellulose, have demonstrated the ability to capture virus-like particles with recoveries exceeding 95% [142]. Given the increasing interest in microalgae as platforms for recombinant protein and VLP production, similar purification strategies may be applicable to microalgal systems, although their application in this context remains to be investigated [143].

The stability of the final product is highly sensitive to temperature and drying methods. While antigens are stable at −80 °C, significant degradation can occur at room temperature [144]. Spray drying is a common industrial method for producing consistent powders, but the high temperatures involved can degrade sensitive vaccine components [145]. Lyophilization (freeze-drying) remains the gold standard for preserving antigen structural integrity and bioactivity over long-term storage [146].

## 3. Plant-Derived Compounds as Vaccine Adjuvants

Plant-derived compounds represent a structurally diverse and biologically active reservoir of molecules with significant immunomodulatory potential. Beyond their traditional roles in phytotherapy and natural product research, many of these metabolites are now being investigated as functional components in modern vaccine formulations. Their ability to engage innate immune pathways and modulate adaptive responses provides a mechanistic foundation for their consideration as next-generation adjuvants.

### 3.1. Introduction and Immunological Rationale

Adjuvants are immunologically active components that increase vaccine efficacy by enhancing, directing, and stabilizing the immune response induced by the antigen [147]. In many recombinant vaccines, the antigen alone is insufficient to activate innate immunity and induce germinal centers, affinity maturation, and durable B and T cell memory; many modern adjuvants act by engaging innate sensors, such as pattern recognition receptors (PRRs), and by shaping local inflammation and antigen trafficking [148].

They optimize antigen transport to draining lymph nodes, increase uptake by APCs and directly activate innate sensors such as Toll-like receptors (TLRs), C-type lectin receptors (CLRs), and NOD-like receptors (NLRs) [149].

Additionally, they create a controlled local inflammatory microenvironment at the administration site through chemokine/cytokine production, which promotes recruitment and maturation of dendritic cells and monocytes and improves antigen presentation to T lymphocytes [150]. This multi-mechanistic view corresponds with modern conceptual frameworks regarding adjuvant biology and explains why formulation, rather than the adjuvant molecule alone, often determines immune outcomes.

Plant-derived compounds are of particular interest as a reservoir for next-generation adjuvants due to the high phytochemical diversity and the possibility of structural engineering [147]. Some phytochemicals are suited for needle-free vaccines because they can navigate the body’s mucosal barriers using lectins to target M-cells or interact directly with the epithelium, making them effective for intranasal or oral immunization [151].

In general, to understand the efficacy of plant-derived adjuvants, we must look beyond their chemical structures and focus on the specific immune programs they initiate.

This outcome-oriented framework classifies phytochemical adjuvants into five functional categories: PRR agonists that activate innate sensors, membrane-active enhancers that facilitate cytosolic access to antigens, depot-forming scaffolds that enhance antigen retention, mucosal targeting molecules that bypass epithelial barriers, and immunometabolic modulators that regulate inflammation via metabolic signaling [147]. This classification clarifies how different phytochemical adjuvants shape immune outcomes, indicating whether a vaccine raises antibody levels alone or also provides cellular and barrier protection, both of which are crucial for long-term immunity.

### 3.2. Major Classes of Phytochemical Adjuvants

**Figure 1:**
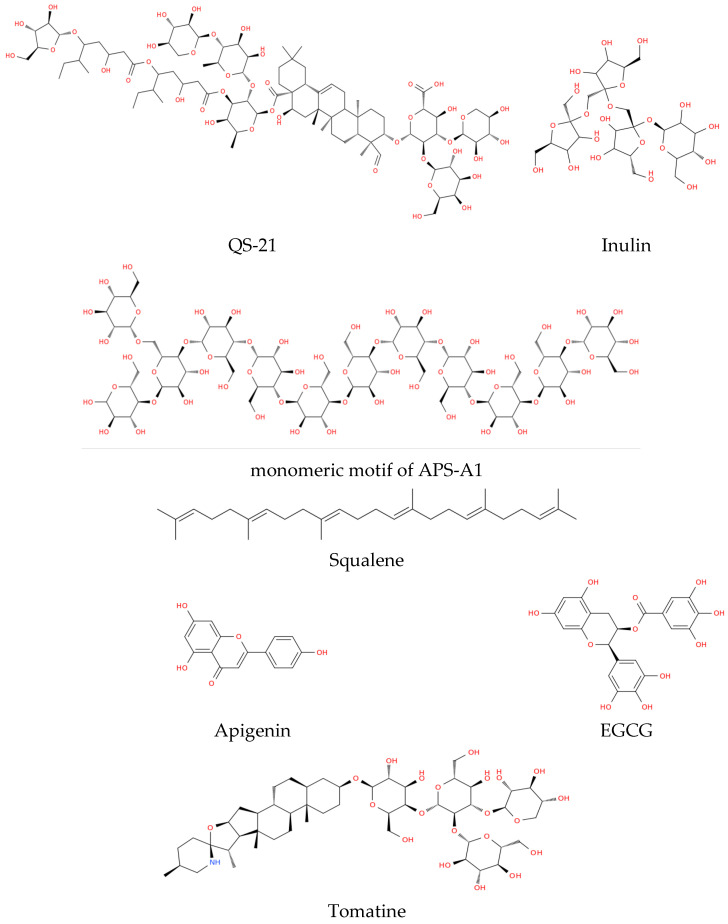
Structural representation of key bioactive molecules discussed in this study, including the triterpenoid saponin QS-21 (one of the components of Matrix-M™), the inulin tetrasaccharide whose higher-order polysaccharide forms constitute the Advax™ adjuvant, and a typical monomeric motif of the *Astragalus* polysaccharide APS-A1, as reported in Ref. [152]. Additional structures shown include squalene, apigenin, epigallocatechin gallate (EGCG), and tomatine.

The phytochemical adjuvants used in vaccines can be broadly categorized into distinct structural and functional groups based on their immunological mechanisms and structural properties (Figure 1). The following subsections outline the principal classes and summarize their roles in contemporary vaccine design.

#### 3.2.1. Saponins, Membrane-Active Enhancers

Saponins are amphiphilic glycosides composed of a hydrophobic aglycone (typically steroidal or triterpenoid) linked to hydrophilic sugar chains. This amphiphilic nature allows saponins to interact with cell membranes and facilitate the translocation of antigens into the cytosol, a process essential for the cross-presentation of antigens to CD8+ T cells. The most important example is QS-21 (purified from *Quillaja saponaria*, Figure 1), which forms the core of several modern adjuvant systems [153]. QS-21, when combined with PRR agonists such as MPL, can generate a rapid innate signature characterized by cytokine production and recruitment of innate effector cells in draining lymph nodes, with an early IFN-γ signal in the lymph nodes serving as a hallmark of the early response. Mechanistically, QS-21 has been linked in multiple models to activation of inflammasome pathways, especially NLRP3, with increased IL-1β/IL-18, although the magnitude of this effect can depend on dose, cell type, and the presence of TLR priming [154].

A key scientific variation is that QS-21 at higher concentrations can induce cell death, and some of this toxicity can be independent of NLRP3; cholesterol-containing contexts can modulate survival and help separate adjuvant activity from toxicity. To lower these risks, QS-21 is stabilized in lipid-based structures, such as liposomes or cholesterol-bound ISCOM-like assemblies [155].

QS-21 is the core component of the AS01 adjuvant system, which is utilized in high-efficacy licensed vaccines such as Shingrix (herpes zoster), Mosquirix (malaria), and Arexvy (respiratory syncytial virus) [156]. The AS01 system is a liposomal formulation that combines 50 μg of QS-21 with 50 μg of monophosphoryl lipid A (MPL), a TLR4 agonist, per 0.5 mL dose in the Shingrix vaccine. In Arexvy, the dosage is adjusted to 25 μg of each component [157]. In addition, the spike ferritin nanoparticle (SpFN) vaccine against COVID-19 represents another QS-21–containing adjuvant system. This vaccine is formulated with Army Liposomal Formulation QS-21 (ALFQ), a liposomal adjuvant comprising QS-21 and a synthetic monophosphoryl lipid A analog (3D-PHAD^®^, a TLR4 agonist). In this system, QS-21 improves antigen-specific immune responses, while the MPL analogue promotes innate immune activation via TLR4 signaling, and the liposomal formulation enhances antigen delivery and decreases systemic reactogenicity. Preclinical and early clinical studies have shown that this combination induces strong neutralizing antibody responses [158].

The cooperation between MPL and QS-21 is critical; MPL activates the innate immune system via the TRIF-dependent signaling pathway, while QS-21 triggers lysosomal destabilization and activates the tyrosine kinase SYK, leading to the assembly of the NLRP3 inflammasome and the subsequent release of pro-inflammatory cytokines such as IL-1β and IL-18 [159]. This combined activation induces a strong IFN-γ response in the draining lymph nodes, which is essential for driving a Th1-biased helper T-cell response and high-affinity antibody production [155].

Saponins are often combined into nanoparticle platforms to optimize delivery and minimize toxicity. The Matrix-M™ adjuvant, used in the Nuvaxovid COVID-19 and R21 malaria vaccines, consists of 40 nm nanoparticles formed by combining Quillaja saponins (fractions A and C) with cholesterol and phosphatidylcholine [160]. This configuration separates the saponin, removes its hemolytic activity, and preserves its ability to recruit dendritic cells (DCs) and support a balanced Th1/Th2 immune response [161]. Likewise, Immunostimulating Complexes (ISCOMs) and ISCOMATRIX are cage-like assemblies that hold antigens with saponins and lipids [162]. Through co-delivery of antigen and adjuvant to an individual APC at the same time, these systems improve antigen processing and presentation through both the MHC I and MHC II pathways [163].

Despite its potency, the QS-21 supply chain is vulnerable because it relies on harvesting wild *Quillaja saponaria* bark, which raises environmental concerns. QS-21 is also chemically unstable due to its two hydrolytically sensitive ester moieties, which may complicate long-term storage and formulation [164].

Moreover, in comparative terms, aluminum salts (alum) are the most widely used adjuvants because of their long safety record, low cost, and strong ability to amplify antibody responses [165]. However, alum is often biased toward Th2-skewed humoral immunity and is comparatively limited in driving robust Th1 responses, potent Tfh/GC programs, and CD8+ T-cell priming, features that are frequently required for modern recombinant subunit vaccines and for pathogens where neutralizing antibodies alone are insufficient [166]. In contrast, QS-21-type saponins, especially when embedded in adjuvant systems (e.g., liposomes with complementary PRR agonists), are more effective at eliciting a Th1-prone signature and improve Tfh/GC activity and support cross-presentation-linked CD8 priming, even though they often increase reactogenicity and narrow formulation tolerability [167]. Thus, the advantage of QS-21 over alum is not a universal superiority, but rather a different immunological operating point, with stronger cellular/Th1/Tfh biology at the expense of greater formulation complexity and tighter safety/CMC constraints [168].

In addition, there are some eco-friendly alternatives from the seeds of *Momordica cochinchinensis*, a tropical plant common in Southeast Asia. Researchers have synthesized VSA-2 (C_94_H_147_NO_42_), a semi-synthetic derivative of *Momordica* saponin II, which contains an amide side chain at the C3 position of its trisaccharide domain. In preclinical models of SARS-CoV-2 subunit vaccines, VSA-2 induced higher neutralizing antibody titers and Th1-prone T-cell responses than QS-21 [169].

Its chemical stability and scalable production make it a favorable candidate to replace *Quillaja*-derived saponins in future vaccine generations.

#### 3.2.2. Polysaccharides, Depot-Forming Scaffolds and PRR Agonists

Plant-derived polysaccharides act as self-adjuvanting delivery vehicles, which provide a physical scaffold for the antigen while simultaneously engaging PRRs [170].

Advax™ is a highly characterized adjuvant derived from δ-inulin, a semi-crystalline β-D-(2-1)-polyfructose sourced from *Dahlia* tubers. Advax is unique among adjuvants because it does not appear to operate through traditional inflammatory danger signaling, such as the activation of the NLRP3 inflammasome; instead, its potency comes from behaving more like an immunological amplifier and delivery scaffold than a stand-alone inflammatory stimulus [171].

Mechanistically, δ-inulin microparticles (∼1–2 μm) are phagocytosed by macrophages and dendritic cells, which increase antigen capture and improve MHC-II presentation and cross-presentation. In clinical trials of hepatitis B vaccines, people who received 5–10 mg doses of Advax showed higher seroprotection rates and antibody geometric mean titers (GMTs) compared to those who received the antigen alone. Advax has also been used in seasonal influenza vaccination for older people, where it increased plasmablast responses and IgG levels [172]. Recent research has evaluated the efficacy of Advax-CpG combinations for COVID-19 and influenza, suggesting improved immune responses and supporting their potential application in overcoming mucosal immune tolerance [173].

Polysaccharides from *Astragalus membranaceus* roots (APS) have been used since antiquity in traditional medicine to improve immunity [174] and are currently investigated as modern vaccine adjuvants. Mechanistically, APS primarily binds to TLR4 on macrophages and dendritic cells and forms a signaling cascade involving MyD88, TRAF6, and NF-κB. This activation causes the secretion of Th1-type cytokines such as IL-12 and IFN-γ, which drives the differentiation of CD4+ T cells toward a protective phenotype [175,176].

Transcriptomic analyses of APS-adjuvanted vaccines show that APS improved the activation of germinal center (GC) B cells and T follicular helper (Tfh) cells, and provided a long-lasting immune protection [177].

In models of SARS-CoV-2 subunit vaccines, the combination of APS with traditional aluminum salts has shown strong synergy, enhancing IgG isotype switching and increasing antibody titers by 1.7-fold compared to alum alone [174]. While polysaccharides are safe and easy to work with, their clinical translation is limited because they are difficult to standardize and their mechanisms are not always clear across different preparations.

#### 3.2.3. Flavonoids and Phenolics, Immunometabolic Modulators

Flavonoids and phenolics are plant metabolites known for their antioxidant and anti-inflammatory properties. Remarkably, some flavonoids, like apigenin and those derived from *Epimedium*, activate the immune system by mimicking signals for viral detection [178]. These compounds also engage TLR7/8, in addition to triggering the NF-κB pathway, which is the main switch for inflammation. This mechanism leads to dendritic cell maturation and increases the expression of co-stimulatory molecules like CD80 and CD86 [179].

Experimental data from murine models showed that purified EGCG (epigallocatechin gallate) from green tea offered a more compatible immune response than traditional aluminum adjuvants, which induced a more balanced Th1/Th2 response with reduced reactogenicity [180].

Apigenin has also been shown to modulate the PI3K/Akt/mTOR and MAPK/ERK signaling pathways, which are essential for controlling cancer cell proliferation and regulating the immune microenvironment [181]. Plant phenolics also serve as aromatic haptens, and due to their highly hydroxylated nature, they can interact non-covalently with proteins, and under oxidative conditions may form covalent adducts. This interaction provides molecular rigidity to the antigen, avoiding premature degradation and enhancing its immunogenicity by presenting a more stable target for B-cell receptors [182].

The main challenge with flavonoids is that their effects are unpredictable. A compound that works as an anti-inflammatory antioxidant in a supplement could act as a pro-inflammatory adjuvant in a vaccine. This means one property does not guarantee the other. To use them in vaccines requires refined formulation strategies to overcome the inherent problem in solubility and systemic pharmacokinetics [183].

#### 3.2.4. Terpenoids, Emulsion-Based Adjuvant Systems

Terpenoids, particularly the triterpene squalene (C_30_H_50_, Figure 1), are foundational to the development of oil-in-water (o/w) emulsion adjuvants. Systems such as MF59^®^ and AS03 have been used in hundreds of millions of doses of pandemic and seasonal influenza vaccines, where they facilitate dose-sparing and broaden the immune response across different viral strains [184]. Emulsion adjuvants generally act in the immunocompetent environment of the injection site instead of a single receptor. MF59 triggers the local release of chemokines such as CCL2 and CXCL8, which recruit neutrophils, monocytes, and dendritic cells to the area. These cells capture the antigen and transport it to the draining lymph nodes for presentation to lymphocytes. AS03 also includes alpha-tocopherol (Vitamin E), which acts as an antioxidant and further increases the immune response, resulting in the generation of long-lived plasma cells [185].

Generally, oil-in-water emulsions such as MF59 and AS03 have a similar design logic. They work by altering the local immunocompetent environment at the injection site, which helps recruit innate cells and improve antigen acquisition and trafficking to draining lymph nodes, rather than functioning as single-receptor agonists [186]. However, they may vary in immune phenotype and reactogenicity due to compositional changes that alter the inflammatory and metabolic characteristics of early innate responses. MF59 is considered an APC recruiter with strong antibody enhancement and breadth [187].

As mentioned earlier, AS03 includes α-tocopherol in addition to the emulsion components, which can further modulate innate activation and immunometabolic pathways, often translating into stronger responses in settings such as dose-sparing or immunologically challenging populations [188]. These differences indicate a vital principle relevant to phytochemical adjuvants. Formulation-level systems biology often outweighs single-molecule identity in determining clinical immune outcomes [189].

In the past, squalene was mainly taken from shark liver oil, which threatens marine biodiversity and is inefficient; it takes between 2500 and 3000 sharks to produce just one ton of product [190]. However, industry is transitioning toward more sustainable sources, such as plant-based or bioengineered squalene, to decrease environmental impact. For example, PhytoSquene^®^, derived from amaranth oil (*Amaranthus caudatus*), offers a non-animal-derived squalene that meets strict pharmaceutical quality standards [191]. Although PhytoSquene^®^ itself is not yet used in licensed vaccines, squalene is already employed in approved adjuvants such as MF59, as discussed above, which is used in licensed influenza vaccines such as Fluad^®^ and Fluad Quadrivalent^®^ [192]. Similarly, AS03 is utilized in pandemic influenza vaccines, including Pandemrix^®^ and Arepanrix^®^ [193].

#### 3.2.5. Alkaloids, Cytosolic Delivery and Modulators

Tomatine (Figure 1) is a glycoalkaloid found in the stems and leaves of tomato (*Solanum lycopersicum*) plants, and has recently been investigated preclinically as an adjuvant component in nanoparticle and mRNA vaccine contexts. Recent studies on SARS-CoV-2 suggest that combining tomatine with lipid nanoparticle (LNP) formulations may enhance humoral and cellular responses. This approach can make mRNA more stable and facilitate cytosolic release [194].

Alkaloids such as berberine and piperine are recognized for their capacity to modulate key signaling pathways in cancer cells, such as mTOR, MAPK, and NF-κB. Berberine has shown synergistic effects when combined with classical cytostatic agents and may prevent tumor immune escape in colorectal and gastric cancers [195]. Piperine, derived from black pepper, enhances the bioavailability of many therapeutics and adjuvants by inhibiting hepatic metabolism and improving intestinal absorption [196]. In general, alum mostly induces Th2-type responses, which are relatively weak in stimulating cellular immunity. In contrast, these alkaloids offer various benefits, such as the capacity to modulate several intracellular signaling pathways, enhance the bioavailability of co-administered agents, and may induce more balanced immune responses, in addition to the largely Th2-biased effects associated with alum.

However, a major drawback of many alkaloids is their narrow therapeutic window. Because these compounds evolved as plant toxins to deter herbivores, the difference between a beneficial immune-enhancing dose and a toxic dose can be minimal.

#### 3.2.6. Lectins, Mucosal Targeting Ligands

Lectins target mucosal surfaces and help antigens reach inductive sites more easily, and promote local secretory immunoglobulin A (sIgA) responses in addition to tissue-proximal immunity [197]. This localized recruitment is particularly critical for the success of needle-free immunization strategies, such as nasal or oral delivery, where overcoming the mucosal barrier is the fundamental challenge to achieving protective immunity.

ArtinM, a D-mannose-binding lectin derived from jackfruit seeds (*Artocarpus integrifolia*), can promote Th1-prone responses by interactions with glycosylated receptors and innate signaling modules in APCs. This interaction increases IL-12 production and strengthens the immune system more than other plant lectins [198]. However, its application is currently limited to preclinical studies, and it has not yet been approved for clinical use.

UEA-1 (*Ulex europaeus* agglutinin-1) can be used for needle-free delivery due to its ability to bind to α-L-fucose residues that are found on M cells. This facilitates transcytosis across mucosal barriers and makes mucosal antibodies stronger [199]. Similarly, UEA-1 remains an experimental tool and is not yet used in routine clinical practice.

Moreover, the clinical application of lectin-based strategies is limited by concerns about mitogenicity, potential allergenicity, and the risk of developing anti-lectin neutralizing antibodies. To reduce side effects, researchers use lectin-derived functional peptides that keep their targeting power without triggering toxic cell growth, ensuring the treatment hits the right cells while avoiding the off-target immune reactions that cause unwanted inflammation [197].

#### 3.2.7. Plant Viruses as Vaccine Components and Adjuvants

In addition to their established role as expression platforms for recombinant antigens and therapeutic proteins, plant viruses themselves represent an emerging class of vaccine components and adjuvants.

Plant viruses have a unique combination of biosafety, structural uniformity, low reactogenicity, and cost-effective production, which makes them attractive scaffolds for next-generation vaccines [200]. Their utility goes beyond antigen display. Several plant viruses and virus-derived nanoparticles have demonstrated intrinsic adjuvant properties, increasing both the magnitude and durability of immune responses. Among these, structurally modified derivatives of Tobacco mosaic virus have proven promising [201].

Spherical particles generated by thermal remodeling of the Tobacco mosaic virus coat protein exhibit this trend with these spherical particles combining a high adsorption capacity, which permits efficient antigen loading, with strong immunostimulatory activity. Preclinical studies have indicated that these molecular biosystems can potentiate immune responses to a variety of antigens in animal models, supporting their designation as plant-virus–based adjuvants [202]. Importantly, comprehensive toxicological evaluations in mice, rats, and rabbits have demonstrated an absence of adverse local or systemic effects following single or repeated administrations. Parameters, including general health status, hematology, blood chemistry, and reproductive and developmental endpoints, remained within normal ranges [203].

Overall, this evidence supports the view that plant viruses are not only adaptable platforms for antigen presentation but also comprise a distinct category of plant-derived adjuvants. Their structural composition adaptability, immunogenic potential, and proven safety position them as strong candidates for incorporation into recombinant vaccine formulations and cancer immunotherapy strategies [204].

### 3.3. Safety, Toxicity, and Translational Challenges

Plant-derived adjuvants face distinct safety and translational barriers. First, several classes show membrane activity that can manifest as hemolysis or broader cytotoxicity at higher concentrations, creating a narrow therapeutic window where potency and tolerability must be separated by formulation [147]. Second, immunostimulation can be a double-edged sword. Overly strong innate activation may increase reactogenicity, skew cytokine profiles unfavorably, or amplify inflammatory pathology in susceptible hosts [205]. Third, natural-source materials may vary due to plant genetics, growth conditions, extraction methods, and impurities that are also extracted. Fourth, numerous candidates have chemical instability (e.g., hydrolysis-prone motifs) or physicochemical properties (poor solubility, aggregation), thereby requiring more sophisticated delivery systems. Finally, regulatory pathways require strict characterization of identity, purity, and mechanism of action, as well as validated examination that connects composition to immune outcomes [206].

Table 2 summarizes the major classes of plant-derived adjuvants, their botanical sources, immunological mechanisms, and current clinical or developmental status.

## 4. Plant-Based Vaccine Delivery Systems: Focus on Oral and Edible Platforms

Plant-based platforms provide an integrated framework for antigen protection, delivery, and immune activation. Their potential lies not only in biological feasibility but also in practical and translational advantages.

### 4.1. Plant Matrices, Bioencapsulation, and Antigen Stability

Cellulose and lignin are major structural components of the rigid plant cell wall, which largely resists degradation by human digestive enzymes [210]. Consequently, when vaccine antigens are produced inside intact plant cells, the tough plant cell wall protects the antigens as they pass through the acidic, enzyme-rich environment of the stomach. The antigen is essentially shielded until it reaches the intestine. Once in the gut, the resident microbiota can partially degrade plant cell walls (as certain gut microbes produce the necessary cellulolytic enzymes), enabling the release of the antigen into the intestinal lumen. There, the antigen can be sampled by the gut-associated lymphoid tissue (GALT), particularly via specialized epithelial M cells in Peyer’s patches, to promote mucosal and, in some cases, systemic immune responses. As this biological encapsulation is inherent to the plant cell wall, researchers have used it to create oral pills or powders by freeze-drying plant tissues. For instance, lyophilized lettuce cells expressing vaccine candidates, such as the protective antigen (PA) from *Bacillus anthracis*, have been shown to retain antigenicity at room temperature and to induce antigen-specific immune responses in animal models following oral administration [211].

The distinction between dietary proteins and vaccine antigens in orally delivered plant-based systems relies on the mechanisms of mucosal immune recognition. Antigens released in the intestinal lumen are sampled by specialized microfold (M) cells [212] and presented to antigen-presenting cells within the GALT [213]. Then, the immune response depends on how the antigen is presented, including its structure, dose, and the presence of immunostimulatory signals. If these signals are insufficient, oral exposure may give rise to immune tolerance, not protective immunity [214].

Collectively, these features may reduce the need for antigen purification and cold-chain storage. Traditional vaccine production in microbial or mammalian cell systems requires purification of the antigen, refrigeration, and sterile delivery, all of which add cost and complexity [215]. In contrast, using edible plant tissues for vaccine production enables a simpler process in which the harvested tissue can be directly formulated into an oral dose, such as a capsule, tablet, or powder [216]. The plant’s storage organelles often naturally compartmentalize the antigen. For example, rice seeds accumulate vaccine antigens in endosperm storage organelles known as protein bodies. The cholera vaccine MucoRice-CTB is a clear example. Transgenic rice grains produced the cholera toxin B subunit, which was deposited in protein bodies that protected it from stomach acid and facilitated its delivery to gut mucosal tissues. Because of this matrix, MucoRice-CTB powder can be stored and transported at room temperature without refrigeration [64].

### 4.2. Examples and Progress in Plant-Based Vaccine Delivery

Overall, oral plant-based vaccines have made significant progress in preclinical and early clinical studies [217]. Human trials using transgenic plants, including potato-based vaccines targeting Norovirus and enterotoxigenic *Escherichia coli*, have confirmed the feasibility of inducing mucosal immune responses through oral immunization [218]. Among the most advanced examples, seed-based platforms such as MucoRice-CTB have shown clinical safety, stability, and immunogenicity in early clinical testing [217]. In addition, some plant-derived vaccine formulations, especially certain freeze-dried preparations, have shown improved stability at ambient temperature [219].

Over the past two decades, early human studies demonstrated the feasibility of potato-derived vaccines for Norovirus and diarrheagenic *E. coli*. In these trials, volunteers ingested doses of transgenic potato tubers expressing the Norwalk virus capsid protein (NVCP) or the *E. coli* heat-labile enterotoxin B subunit (LT-B) [107,117]. This led to an increase in antigen-specific mucosal IgA antibodies, indicating that the oral immunization triggered mucosal immunity [220]. Although the need to consume raw potatoes and challenges in dose standardization were major drawbacks, these studies showed that an antigen could be delivered orally via a food plant and elicit an immune response. Following that, a variety of plant-based vaccine strategies have been explored, including leafy greens (for instance, lettuce expressing hepatitis B antigen, given in a salad or freeze-dried form) and even fruits like bananas (conceptualized for vaccine delivery, though issues of post-harvest variability and dose standardization have made fruits less practical for standardized vaccine delivery) [107,221,222].

Plant-based vaccines have demonstrated promise in veterinary medicine. A notable case is the corn-based oral vaccine for pigs against Transmissible Gastroenteritis Virus (TGEV, a coronavirus). Researchers engineered corn to produce a TGEV antigen in its kernels; feeding this vaccine-enriched corn to sows elevated their anti-TGEV antibody levels and conferred passive protective immunity to piglets via maternal antibodies in milk. The dried corn grain efficiently delivered the antigen and could be administered via regular feed, decreasing the need for injections [220].

Oral vaccine delivery has benefits for enteric infections, as it directly targets the mucosal immune system of the gastrointestinal tract [223]. Since many pathogens, such as Norovirus, enterotoxigenic *E. coli*, and TGEV, initiate infection at mucosal surfaces, the induction of local IgA responses through oral immunization can provide a first line of defense at the site of pathogen entry. In contrast, parenteral vaccines often induce strong systemic immunity but may be less effective at inducing mucosal immune responses [224]. However, oral vaccines also face challenges, including antigen degradation in the gastrointestinal environment and the risk of inducing oral tolerance rather than protective immunity [225].

Despite these advances, oral plant-based vaccines have limitations. A significant challenge is their weak and variable immunogenicity, which often leads to insufficient immune activation compared to parenteral vaccines [226]. After oral administration, antigens are exposed to harsh gastrointestinal conditions, including acidic pH and digestive enzymes, leading to partial degradation and reduced bioavailability [227]. In addition, oral delivery may induce immune tolerance, particularly in the absence of adequate immunostimulatory signals [228].

To address these limitations, a variety of strategies have been implemented to strengthen the immune-stimulating capacity of plant-derived oral vaccines. For instance, bioencapsulation in plant cells protects antigens from gastrointestinal degradation and improves delivery to GALT [217]. The use of mucosal adjuvants, including plant-derived compounds and carrier molecules such as CTB or LTB, can enhance immune activation [229]. Another strategy involves targeting antigen delivery to M cells in Peyer’s patches to enhance mucosal sampling [230,231]. In addition, increasing antigen expression levels through optimized constructs, particularly chloroplast-based systems, can help overcome dose limitations [232]. Integrating oral and parenteral immunization in a prime-boost format can also strengthen and sustain immune responses [233].

### 4.3. Advantages, Challenges, and Limitations

Plant-based vaccines offer potential benefits for pharmaceutical applications [234]. They can be administered as oral capsules with food or in beverages to improve patient compliance [215]. Additionally, they decrease the risks related to needles, including infections, pain, and potential administration errors [235]. This approach decreases the need for expensive purification, cold-chain storage, sterile injections, and complex manufacturing systems [215,236]. Freeze-dried plant material is stable at room temperature and can be produced at a large scale, making this approach a low-cost alternative to conventional injectable biopharmaceuticals [237]. Moreover, production relies primarily on plant materials, thereby reducing the risk of contamination by adventitious agents (e.g., adventitious viruses) or prions associated with animal cell-based manufacturing [238].

This strategy also faces several important challenges before widespread use. Achieving sufficiently high and consistent antigen expression is important, as low expression would require impractically large doses; although chloroplast engineering improves yields, each antigen-plant system often needs specific optimization [53,239]. Compared with purified injectable vaccines, maintaining accurate dose uniformity and regulatory compliance is more difficult, requiring additional good manufacturing practice-compliant processes to ensure batch-to-batch consistency and quality control [236]. Also, oral delivery may induce immune tolerance rather than protection [240]. Finally, long-term antigen stability must be validated for each product, and regulatory and public acceptance issues related to genetically modified organisms (GMOs) remain key hurdles [241].

Despite significant progress, several limitations remain. Much of the evidence supporting plant- and microalgae-based vaccine platforms is still preclinical, with limited late-stage clinical validation [93]. Variability in expression levels, challenges in large-scale Good Manufacturing Practice (GMP) standardization, glycosylation differences, and potential risks such as oral tolerance may affect translational consistency and immunogenicity [217]. For phytochemical adjuvants, structural heterogeneity and incomplete mechanistic characterization complicate standardization and regulatory approval [242]. Continued clinical evaluation and industrial-scale validation are therefore essential to confirm the practical feasibility of these platforms [93].

## 5. Regulatory Considerations and Translational Challenges

Despite all the progress in plant-based vaccine development, regulatory approval is still limited because of strict requirements imposed by major agencies, including the U.S. FDA, the European Medicines Agency (EMA), and Health Canada. To date, one example is the plant-derived COVID-19 VLP vaccine (CoVLP, Covifenz^®^), which received authorization for human use in Canada, although its commercial development was later discontinued [93]. No plant-based vaccines have yet to receive full approval from the FDA or the EMA, which reveals the regulatory barriers to broad adoption [243].

Regulatory systems for plant-derived vaccines generally follow the same principles as those for conventional biologics and require strict adherence to GMP, along with detailed characterization of product identity, purity, potency, and safety [244]. However, plant-based systems add complexities. Variability in antigen expression across plant hosts, growth conditions, and production clusters can complicate standardization and reproducibility. Plant-specific post-translational modifications, especially non-human glycosylation patterns, must also be evaluated for their effects on immunogenicity and safety [96,245].

Downstream processing and purification also offer regulatory complications related to the removal of host cell proteins, DNA, plant metabolites (e.g., phenolics and alkaloids), and potential contaminants. For orally delivered plant-based vaccines, additional concerns include dose standardization, stability, and control of antigen release, since whole plant tissues are used as delivery matrices instead of purified formulations [246].

Another important regulatory consideration involves biosafety and environmental containment, especially for genetically modified plants. Regulatory agencies need strict methods to prevent unintended gene flow, cross-contamination with food crops, and environmental release [96]. General approval of GMOs also affects regulatory pathways and market adoption [247].

Finally, the lack of harmonized international guidelines specific to plant-made pharmaceuticals (PMPs) is a principal limitation. While agencies such as the FDA and EMA have issued general guidance for biologics and recombinant proteins, dedicated regulatory structures designed for plant-based systems are still evolving [248].

## 6. Conclusions

Vaccination remains one of the most powerful tools in public health, yet its global impact continues to be constrained by challenges in antigen identification, production scalability, cost, and delivery logistics. Traditional manufacturing platforms (particularly mammalian cell cultures and transgenic animals) offer high-quality post-translational processing but are limited by prohibitive costs, complex infrastructure requirements, and difficulties in achieving the large antigen quantities needed for oral immunization. These limitations are especially pronounced for subunit vaccines, which, despite their excellent safety profiles, often require high-yield expression systems and effective adjuvantation strategies to achieve robust immunogenicity. Within this context, plant-based vaccine technologies have emerged as a compelling alternative capable of addressing some of these long-standing barriers. Plants and microalgae function as versatile recombinant biofactories, supporting nuclear, chloroplast, and transient expression systems that can generate diverse antigens. Their inherent scalability, low production costs, and minimal biosafety risks make them particularly attractive for large-volume vaccine manufacturing. Moreover, edible plant tissues and plant-derived matrices offer unique opportunities for oral vaccine delivery, reducing or eliminating purification steps, simplifying distribution, and potentially bypassing cold-chain requirements, factors that collectively enhance accessibility in resource-limited settings. Beyond their role as antigen production platforms, plants also provide a rich source of bioactive metabolites with immunostimulatory and adjuvant properties. Specific phytocompounds have demonstrated the capacity to enhance antigen presentation, modulate innate and adaptive immune pathways, and potentiate vaccine-induced responses. These molecules expand the functional repertoire of plant-based vaccine systems, enabling the development of formulations that combine antigen expression, immune enhancement, and delivery within a single biological matrix. Microalgae further contribute to this landscape by offering rapid growth rates, high protein yields, and the potential for oral or mucosal delivery formats. Together, plant-derived vaccines, microalgae-based antigen platforms, phytochemical adjuvants, and plant-origin delivery systems represent a rapidly advancing field with the potential to transform vaccine accessibility, scalability, and immunogenicity. By integrating recombinant expression technologies with bioactive plant metabolites and innovative delivery strategies, these approaches may provide a flexible and sustainable foundation for future vaccine development, pending further clinical and regulatory validation. Their combined advantages (low cost, ease of scale-up, biosafety, and compatibility with oral administration) make plant-based systems promising candidates for further investigation toward addressing global immunization needs, particularly in regions where traditional vaccine production and distribution remain challenging. As research continues to refine expression efficiency, antigen stability, phytochemical characterization, and delivery mechanisms, plant-based technologies are poised to play an increasingly central role in the design of future vaccines.

Despite these advantages, some important drawbacks must be considered. The variability of antigen expression across different plant systems can complicate standardization and reproducibility on a wide scale under GMP settings. In addition, plant-specific post-translational modifications, particularly non-human glycosylation patterns, may affect antigen functionality, immunogenicity, or safety. For oral and edible vaccines, further challenges include the risk of inducing immune tolerance rather than protective immunity, as well as difficulties in achieving precise dose control when using whole plant tissues as delivery matrices. Issues such as product consistency, stability, biosafety, and public acceptance of GMOs continue to limit progress, highlighting the need for improved technologies and clearer regulatory frameworks.

Multiple scientific, manufacturing, and regulatory factors contribute to the limited number of licensed plant-based vaccines. The main challenge lies in translating successful experimental results into scalable, regulatory-compliant manufacturing systems. Key bottlenecks include achieving consistent, GMP-compliant production in inherently variable plant-based systems and standardizing doses, especially for oral formulations. In addition, plant-specific post-translational modifications and product heterogeneity introduce uncertainties, complicating regulatory evaluation. Compared with more established platforms, such as mammalian cell-based or mRNA technologies, plant-based systems still lack well-defined and harmonized regulatory pathways. To support the wider use and market development of plant-based vaccines, it will be essential to address broader systemic and translational challenges rather than focusing solely on improving antigen expression.

## Figures and Tables

**Table 1:** Overview of representative plant-based vaccine candidates, including edible (oral) and non-edible platforms, highlighting pathogen target, antigen type, plant expression host, production strategy, and clinical or regulatory status. It is important to note that only a limited number of candidates have reached advanced clinical stages, while the majority remain in preclinical or early clinical development.

Pathogen/Disease Target	Antigen/Product Description	Plant Host/ Expression Organism	Expression Technology/ Method	Platform Type	Regulatory Status/ Use in Humans	References
SARS-CoV-2 (COVID-19)	Spike (S) protein VLP (CoVLP/Covifenz^®^)	*Nicotiana benthamiana*	Transient expression (*Agrobacterium*-mediated agroinfiltration)	VLP	Authorized for human use in Canada (Covifenz^®^); authorization was later canceled by the sponsor (31 March 2023)	[103]
Influenza (Seasonal & Pandemic)	Hemagglutinin (HA)/Quadrivalent Virus-Like Particles (QVLP)	*Nicotiana benthamiana*	Transient expression (*Agrobacterium*-mediated)	VLP	Advanced Clinical Trials—Phase III completed (efficacy trials conducted)	[104,105]
Human Papillomavirus (HPV)	L1 capsid protein VLPs	*Nicotiana benthamiana*	Transient expression	VLP	Preclinical/Early clinical	[106]
Norovirus	Norwalk virus capsid protein (NVCP)	Potato *(Solanum tuberosum)*	Stable transformation (nuclear)	Edible/Oral	Early human trials (oral immunogenicity)	[107]
Hepatitis B	Hepatitis B surface antigen (HBsAg) expressed in plants	Potato (*Solanum tuberosum*), Lettuce (*Lactuca sativa*, edible); *Nicotiana tabacum* (non-edible research host)	Stable transformation (nuclear)	Edible/Oral	Early clinical studies (Phase I/II oral immunogenicity), but no licensed product	[108]
Cholera	Cholera toxin B subunit (CTB) expressed in MucoRice-CTB (rice)	*Oryza sativa* (rice) MucoRice-CTB	Stable transformation (seed-based)	Edible/Oral	Phase I clinical trial (oral delivery) completed	[109]
Rotavirus	VP6/VP7 antigens (plant-expressed)	*Nicotiana* spp.	Transient/stable expression	Subunit	Preclinical	[110,111]
Rabies	Rabies virus glycoprotein (G protein) (plant-produced antigen/chimeric constructs)	Spinach (*Spinacia oleracea*, chimeric plant virus vaccine), *Nicotiana benthamiana*, *Nicotiana tabacum*	Transient expression & stable/transgenic expression approaches	Subunit/Oral	Preclinical/Early human exploratory immunogenicity (Phase I-like oral feeding studies)—no licensed product	[112,113,114]
Ebola Virus	Ebola Immune Complex (EIC) based on Ebola glycoprotein GP1 fused to an antibody scaffold	*Nicotiana benthamiana*	Transient expression (geminiviral replicon vectors)	Immune complex	Preclinical—immunogenic in mice; plant system also used for ZMapp mAbs (therapeutic antibodies), but no licensed plant-derived vaccine	[115]
Dengue Virus	Envelope protein (E)/domain III constructs	*Nicotiana benthamiana*	Transient expression	Subunit	Preclinical	[116]
Enterotoxigenic *E. coli* (ETEC)	Heat-labile enterotoxin B subunit (LTB)	Transgenic maize (*Zea mays*), Transgenic potato (*Solanum tuberosum*)	Stable transformation (nuclear)	Edible/Oral	Early human oral immunogenicity studies (Phase I-like/exploratory)—no licensed product	[117]
HIV	gp120/gp140 glycoproteins	*Nicotiana benthamiana*	Glycoengineered transient expression	Subunit	Preclinical	[118]
Veterinary (TGEV)	Coronavirus antigen (TGEV)	Transgenic maize	Stable expression (seed-based)	Edible (feed-based)	Veterinary use (preclinical/field studies)	[119]

**Table 2:** Major classes of plant-derived adjuvants, their botanical sources, immunological mechanisms, and current clinical or developmental status. It should be noted that these classes differ in their level of clinical validation, with saponin- and emulsion-based systems being the most advanced, while other phytochemical categories remain largely at the preclinical stage.

Category	Primary Example	Botanical Source	Target Receptor/ Mechanism	Clinical Status/ Application	Refs.
Saponins	QS-21	*Quillaja saponaria*	NLRP3 Inflammasome, Syk kinase	Licensed (Shingrix, Mosquirix, Arexvy)	[154]
Saponins	ISCOMs/ISCOMATRIX	*Quillaja saponaria*	Antigen co-delivery, MHC I/II presentation	Clinical/Advanced development	[162,163]
Saponins	Matrix-M™	*Quillaja saponaria*	DC recruitment, Th1/Th2 balance	Licensed (Nuvaxovid, R21)	[160]
Saponins	Quil-A	*Quillaja saponaria*	Membrane permeabilization, immune activation	Preclinical/Veterinary use	[153]
Saponins	VSA-2	*Quillaja saponaria*	Enhanced Th1 response, improved stability	Preclinical (SARS-CoV-2 models)	[169]
Polysaccharides	Advax™ (delta inulin)	*Dahlia variabilis*	Alternative pathway (Non-inflammasome)	Clinical Trials (HBV, Influenza)	[207]
Polysaccharides	APS	*Astragalus membranaceus*	TLR4, MyD88- NF-κB pathway, Dendritic cell maturation	Preclinical (Synergy with Alum)	[208]
Polysaccharides	β-glucans	Various plants/fungi	Dectin-1, complement activation	Preclinical/Immunomodulation	[209]
Terpenoids	Squalene (MF59)	Shark-derived, *Amaranthus caudatus* (Alternative)	Chemokine release (CCL2, CXCL8)	Licensed (MF59, Fluad)	[184,185,186,187]
Terpenoids	Squalene (AS03)	Plant-derived/mixed sources	Enhanced innate activation (α-tocopherol synergy)	Licensed (Pandemrix, Arepanrix)	[185,188]
Flavonoids	EGCG	*Camellia sinensis*	TLR7/8, PI3K/Akt/mTOR	Preclinical/Cancer research	[180,181]
Flavonoids	Apigenin	Various plants	PI3K/Akt/mTOR, MAPK pathways	Preclinical	[181]
Flavonoids	Epimedium flavonoids	*Epimedium* spp.	TLR7/8 activation	Experimental	[179]
Alkaloids	Tomatine	*Solanum lycopersicum*	Membrane disruption, LNP stabilizer	Preclinical (mRNA platforms)	[194]
Alkaloids	Berberine	*Berberis* spp.	NF-κB, mTOR modulation	Preclinical/adjunct therapy	[195]
Alkaloids	Piperine	*Piper nigrum*	Bioavailability enhancement	Experimental	[196]
Lectins	ArtinM	*Artocarpus integrifolia*	IL-12 induction, Th1 polarization	Preclinical	[198]
Lectins	UEA-1	*Ulex europaeus*	M-cell targeting, mucosal delivery	Experimental	[199]

## Data Availability

No new data were created or analyzed in this study. Data sharing is not applicable to this article.

## Abbreviations

The following abbreviations are used in this manuscript:

CaMV	Cauliflower Mosaic Virus
CRISPR	Clustered Regularly Interspaced Short Palindromic Repeats
Cas9	CRISPR-associated Protein 9
RNAi	RNA Interference
UTR	Untranslated Region
ER	Endoplasmic Reticulum
KDEL	Lys-Asp-Glu-Leu (ER retention signal)
VLP	Virus-Like Particle
HPV	Human Papillomavirus
HBcAg	Hepatitis B Core Antigen
CPMV	Cowpea Mosaic Virus
HA	Hemagglutinin
SARS-CoV-2	Severe Acute Respiratory Syndrome Coronavirus 2
APC	Antigen-Presenting Cell
MHC	Major Histocompatibility Complex
DSP	Downstream Processing
HCP	Host Cell Protein
SEC	Size-Exclusion Chromatography
AEC	Anion-Exchange Chromatography
SXC	Steric Exclusion Chromatography
HPH	High-Pressure Homogenization
CO_2_	Carbon Dioxide
PTM	Post-Translational Modification
RBD	Receptor-Binding Domain
ACE2	Angiotensin-Converting Enzyme 2
CTB	Cholera Toxin B Subunit
LTB	Heat-Labile Enterotoxin B Subunit
qPCR	Quantitative Polymerase Chain Reaction
VP1	Viral Protein 1
VP2	Viral Protein 2
VP28	Viral Protein 28
GnTI	N-Acetylglucosaminyltransferase I
Neu5Ac	N-Acetylneuraminic Acid
CMP	Cytidine Monophosphate
Ti plasmid	Tumor-Inducing Plasmid
PRRs	Pattern Recognition Receptors
TLRs	Toll-Like Receptors
CLRs	C-Type Lectin Receptors
NLRs	NOD-Like Receptors
MPL	Monophosphoryl Lipid A
Tfh	T Follicular Helper Cells
GC	Germinal Center
APS	Astragalus Polysaccharides
EGCG	Epigallocatechin Gallate
LNP	Lipid Nanoparticle
sIgA	Secretory Immunoglobulin A
UEA-1	Ulex europaeus Agglutinin-1
GMTs	Geometric Mean Titers
GALT	Gut-Associated Lymphoid Tissue
NVCP	Norwalk Virus Capsid Protein
TGEV	Transmissible Gastroenteritis Virus
GMP	Good Manufacturing Practice
GMOs	Genetically Modified Organisms
